# A radiographic artificial intelligence tool to identify candidates suitable for partial knee arthroplasty

**DOI:** 10.1007/s00402-024-05589-8

**Published:** 2024-10-03

**Authors:** Thomas J. York, Bartosz Szyszka, Angela Brivio, Omar Musbahi, David Barrett, Justin P. Cobb, Gareth G. Jones

**Affiliations:** 1 MSk Lab, Imperial College London, Sir Michael Uren Hub, 86 Wood Lane, London, W12 0BZ UK; 2 Istituto Clinico Citta Studi, Milan, Italy; 3 School of Engineering Sciences, University of Southampton, Southampton, UK; 4 King Edward VII’s Hospital, London, UK

**Keywords:** Knee replacement surgery, Artificial intelligence, Radiographic decision tool, MSK radiography, Machine learning algorithm, Partial knee arthroplasty

## Abstract

**Introduction:**

Knee osteoarthritis is a prevalent condition frequently necessitating knee replacement surgery, with demand projected to rise substantially. Partial knee arthroplasty (PKA) offers advantages over total knee arthroplasty (TKA), yet its utilisation remains low despite guidance recommending consideration alongside TKA in shared decision making. Radiographic decision aids exist but are underutilised due to clinician time constraints.

**Materials and methods:**

This research develops a novel radiographic artificial intelligence (AI) tool using a dataset of knee radiographs and a panel of expert orthopaedic surgeons’ assessments. Six AI models were trained to identify PKA candidacy.

**Results:**

1241 labelled four-view radiograph series were included. Models achieved statistically significant accuracies above random assignment, with EfficientNet-ES demonstrating the highest performance (AUC 95%, F1 score 83% and accuracy 80%).

**Conclusions:**

The AI decision tool shows promise in identifying PKA candidates, potentially addressing underutilisation of this procedure. Its integration into clinical practice could enhance shared decision making and improve patient outcomes. Further validation and implementation studies are warranted to assess real-world utility and impact.

## Introduction

The prevalence of knee osteoarthritis is increasing, and it already affects one quarter of the UK’s population [1]. Knee replacement surgery is a successful treatment option for patients with end-stage symptomatic knee osteoarthritis who have exhausted non-operative measures, and demand is predicted to increase almost 40% by 2060 [2, 3]. In almost half of patients requiring knee replacement surgery, the osteoarthritis is confined to only one of the three knee compartments, making them eligible for a partial knee arthroplasty (PKA), as well as a total knee arthroplasty (TKA). In the appropriately selected patient, partial knee replacement surgery has a number of advantages; this smaller procedure is cost-effective [4], and associated with a faster recovery and more normal gait [5], as well as a significantly lower risk of serious complications such as infection, blood clots, cerebrovascular accidents and death [6].

For this reason, in the UK, the National Institute for Clinical Excellence (NICE) issued guidance in 2020 stating that PKA should be offered to all suitable patients, alongside TKA, together with a discussion of the potential benefits and risks of each procedure as part of a shared decision making process [7]. Unfortunately, this has had no perceptible impact on UK knee replacement practice, with partial knee replacements representing only 9.1% of all primary knee replacements in 2019 prior to the new NICE guidelines [1], and 9.8% in the latest 2023 report [2]. This underutilisation is mirrored in joint registries around the world [8–10].

The implication is that surgeons, the majority of whom infrequently perform PKAs, remain unfamiliar with the concept and indications for partial knee replacement surgery, and so are not identifying patients who might be eligible for this procedure [11]. There is good evidence that surgeons for whom PKAs constitute at least 20% of their knee replacement, and *ipso facto* use a broader selection criteria, achieve better results for their patients [12]. Whereas, the current reality is that for more than half of knee surgeons in the UK, Australian and New Zealand Registries PKAs constitute less than 5% of their practice [11]. This has led to the development of radiographic decision aids to assist low volume PKA surgeons in identifying suitable patients [13, 14], but these aids require clinicians to engage in the concept of partial knee replacement surgery, and then time to complete, which is a barrier to their use.

A possible solution is an automatic tool capable of screening patients’ knee radiographs to determine whether they are PKA candidates. This information could then be provided to the patient, their primary care doctor, and orthopaedic team, in order to facilitate shared decision making. This study set out to ascertain whether a machine learning algorithm could be used to develop a software tool of this nature, capable of emulating the ability of high-volume PKA surgeons to identify potential PKA candidates using routine knee radiographs.

## Material and methods

This retrospective study utilised pre-existing, pseudonymised patient data and did not require participant involvement of any sort. Ethical approval was granted by the National Research Ethics Service (London, UK, REC Reference: 18/CAG/0141).

A dataset of 6000 unique unilateral knee radiographs in Digital Imaging and Communications in Medicine (DICOM) format was acquired from Imperial College Healthcare Trust (ICHT), London, UK. To be included in the dataset, radiographs had to meet the following eligibility criteria:

- Male or female adult patient, at least 18 years of age at the time of imaging
- Imaging requested for suspected or confirmed osteoarthritis of the knee
- No pre-existing knee arthroplasty
- Four-view knee radiographs available (weight bearing anteroposterior, Rosenberg, lateral and Skyline)

The dataset was then reviewed by the members of an expert advisory panel (EAP) made up of three consultant orthopaedic surgeons, subspecialised in knee arthroplasty. All three are high volume PKA surgeons, performing more than 30 PKAs per year, and with PKA constituting more than 20% of their knee replacement practice [12, 15]. They were asked to assign the most appropriate arthroplasty option for each set: no arthroplasty (NA), medial or lateral partial knee arthroplasty (PKA), patellofemoral joint arthroplasty (PFJA), and total knee arthroplasty (TKA). Each surgeon conducted this initial review independently and had no knowledge of how the imaged patient was subsequently managed. Where all three surgeons reached the same conclusion, the image set was labelled with the corresponding arthroplasty option. In the event of the panel assigning different arthroplasty options to the same image set, the radiographs were subsequently reviewed at a virtual meeting and a consensus opinion reached.

To achieve improved interoperability with deep learning software images were converted from DICOM to JPEG format with a file size of 512 × 512 pixels. Images containing bilateral knee joints were split into unilateral images of the left and right knee. Each image set was randomly assigned, by allocation of its corresponding unique identifier, to the training, validation, or test set with 70%, 15%, and 15% probability respectively.

**Fig. 1:**
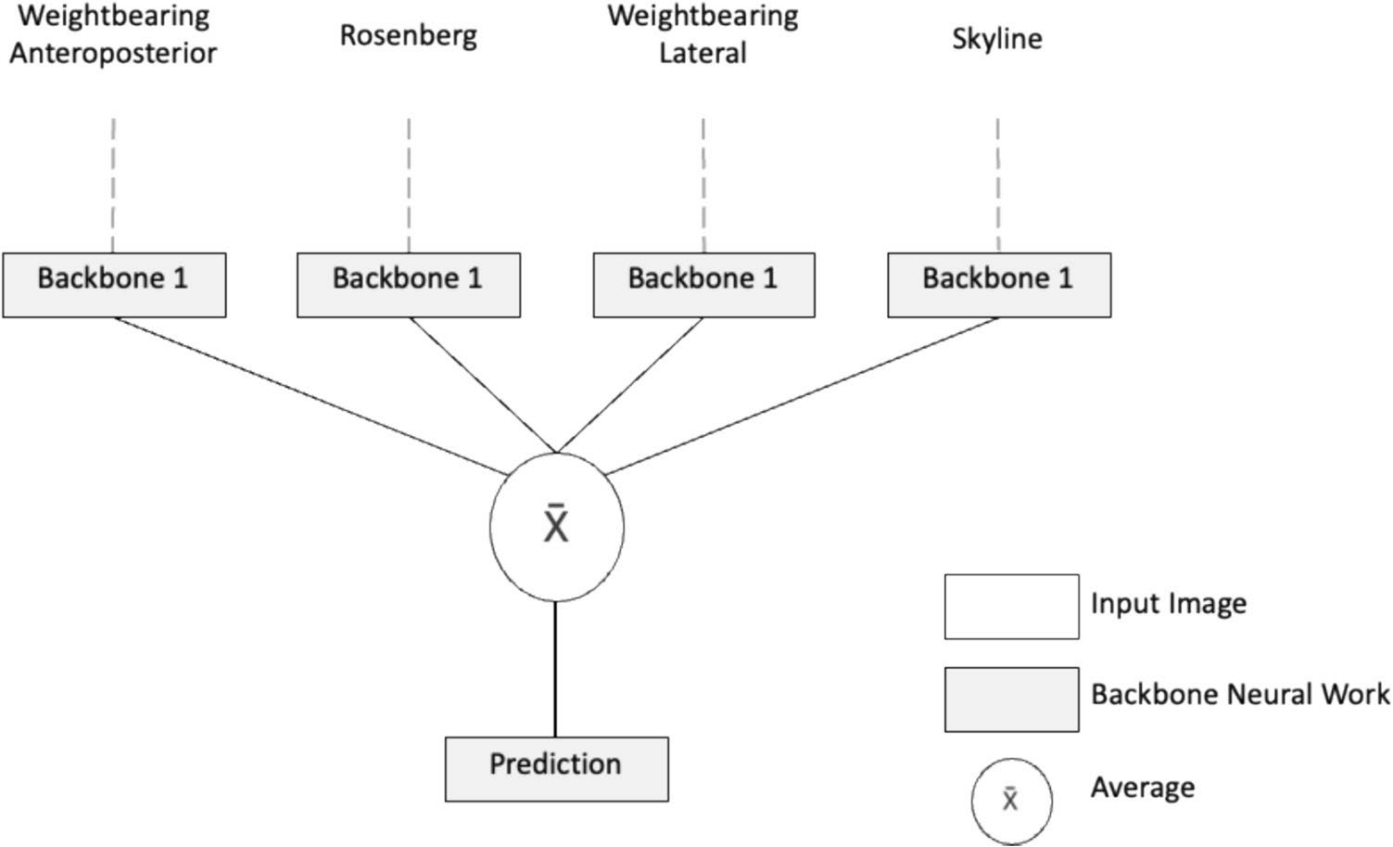
Schema of the architecture used by the neural networks to reach an overall treatment prediction

An exchangeable neural network module was utilised with a model that averaged the outcomes of the assessment of each of the four radiographic views to determine an overall prediction (see Fig. 1).

A total of six machine learning models were assessed for their ability to predict the correct arthroplasty option: Inception v3, ResneXt-TS, EfficientNet-ES, EfficientNet-B2, MobileNet-v2 from *pytorch image models (timm)* library, and Densenet-121 from the *torchxrayvision* library. The models were trained using a standardised training procedure, with further training using cross-entropy with label smoothing (α smooth of 0.05) and class weights to address dataset imbalance. Training occurred with a 16-bit precision and early stop optimisation after 500 steps without improvements in the validation loss. The AdamW optimizer (Adam Optimizer with Nesterov Momentum) was employed for all experiments, with a learning rate of 0.001 chosen along with weight decay of 0.0001. Some models used in the analysis could be started from a pretrained state. Images from the *timm* library were pretrained on ImageNet, a collection of coloured, real images. Images from the *torchxrayvision* library were pretrained on chest radiograph images for pneumonia detection [16].

Model outcomes were assessed on performance for individual class assignment by the calculation of F1 scores (F1), area under the ROC curve (AUROC), and accuracy (Acc).

## Results

**Fig. 2:**
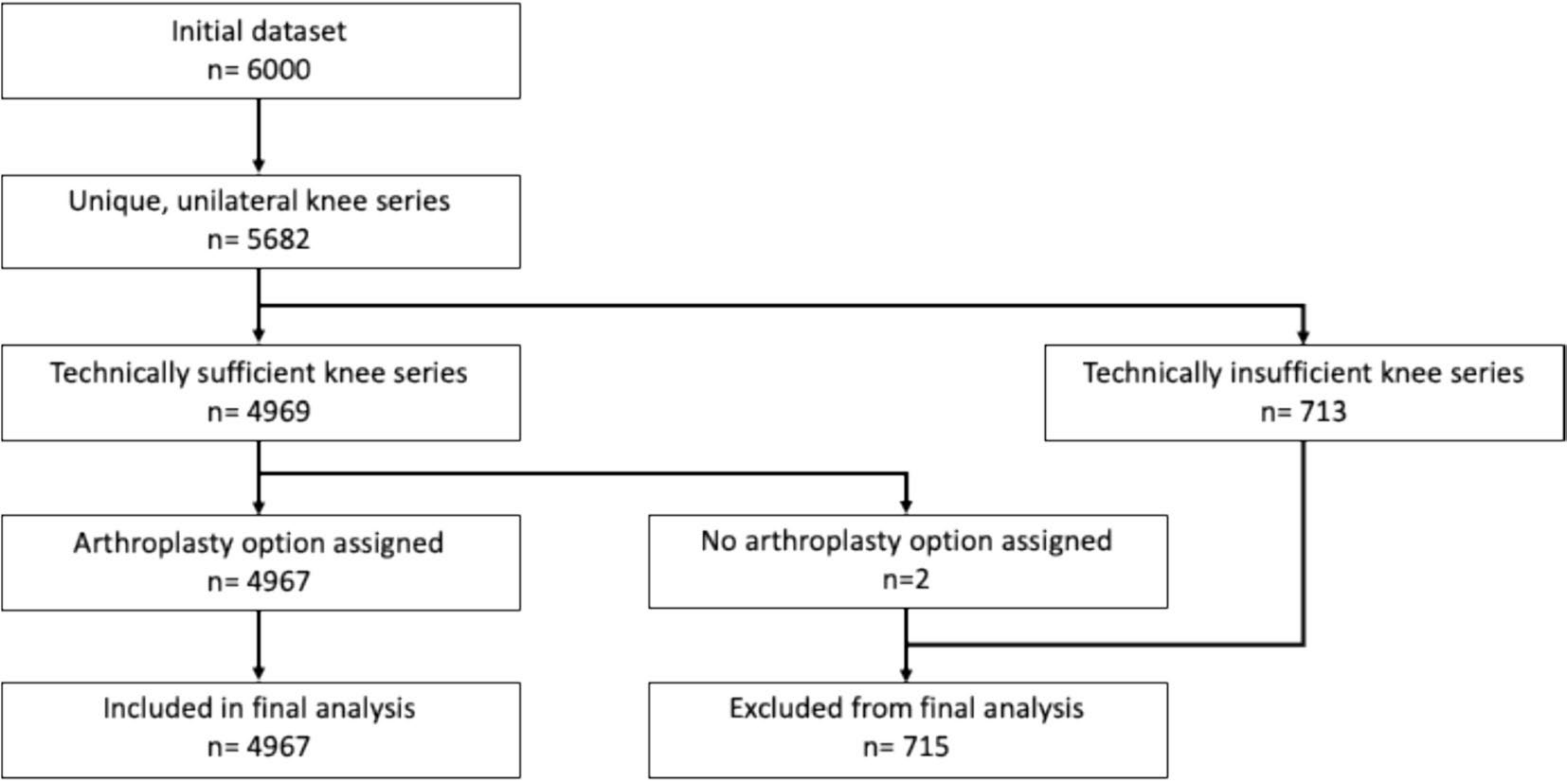
Data processing flowchart

Of the 6000 radiographs acquired from the initial dataset, and following division into imaging for unique unilateral knees, a total of 5682 were assessed as suitable for manual quality control (see Fig. 2). Following the completion of this process, a total of 4969 images (1241 patients) were considered to contain four technically accurate views of the knee, all of these were therefore deemed eligible for final inclusion. Of the total 4969 images, 2509 (50.5%) were left knees and 2460 (49.5%) were right knees. There was agreement amongst all three surgeons in their initial assessment of arthroplasty option for 4630 (93.2%) of the radiographic series. A consensus decision was subsequently reached on 337 of the remaining 339 knee radiographs, with two series (one left and one right knee) excluded due to issues with the images that had not been detected in the initial technical sufficiency review.

Consequently, a total of 4967 images were labelled with an arthroplasty option and progressed to final analysis.

The most commonly chosen treatment option was a partial knee arthroplasty (3284, 56.1%). This was followed by no arthroplasty (792, 15.9%), then total knee arthroplasty (731, 14.7%). Patellofemoral joint arthroplasty was the least commonly indicated surgical management (160, 3.2%) (Table 1).

**Table 1:** A breakdown of the treatment options determined by the expert surgeon advisory panel, in total and divided by laterality

Arthroplasty option	Left	Right	Total (%)
No arthroplasty (NA)	428	364	792 (15.9%)
Partial arthroplasty (PKA)	1613	1671	3284 (56.1%)
Patella-femoral arthroplasty (PFJA)	88	72	160 (3.2%)
Total arthroplasty (TKA)	379	352	731 (14.7%)
All	2508	2459	4967

The mean outcomes for each tested model, covering both left and right lateralities, and including F1 scores, AUROC, and Accuracy (Acc), are reported individually in Table 2*.*

**Table 2:** F1 score (F1), area under the receiver operating curve (AUROC) and accuracy (Acc) results for the different machine learning prediction models for each treatment class i.e. no arthroplasty (NA), partial knee arthroplasty (PKA), patellofemoral joint arthroplasty (PFJA) and total knee arthroplasty (TKA)

Model	F1	AUROC	ACC		F1	AUROC	ACC
DenseNet-121-xray				ResNeXt-26-TS			
NA	0.6365	0.957	0.7915	NA	0.588	0.9625	0.8335
PKA	0.8185	0.944	0.762	PKA	0.7835	0.922	0.718
PFJA	0.25	0.6335	0.3335	PFJA	0.375	0.6945	0.5
TKA	0.4285	0.7645	0.4225	TKA	0.412	0.7475	0.418
EfficientNet-B2A (awts = 0)				EfficientNet-B2A (awts = 0.5)			
NA	0.5995	0.959	0.7915	NA	0.631	0.958	0.8335
PKA	0.818	0.9345	0.7485	PKA	0.813	0.9395	0.7555
PFJA	0.125	0.769	0.1665	PFJA	0.2	0.9325	0.1665
TKA	0.42	0.757	0.4635	TKA	0.4415	0.776	0.25
EfficientNet-B2A (awts = 1)				EfficientNet-ES			
NA	0.6345	0.959	0.7915	NA	0.679	0.954	0.875
PKA	0.8	0.9345	0.7485	PKA	0.8335	0.9515	0.794
PFJA	0.2	0.769	0.1665	PFJA	0.3335	0.785	0.3335
TKA	0.419	0.757	0.468	TKA	0.3425	0.7365	0.3275
Inception v3				MobileNet v2 120d			
NA	0.5545	0.9595	0.667	NA	0.6385	0.963	0.9165
PKA	0.7805	0.9255	0.741	PKA	0.8015	0.931	0.746
PFJA	0.3335	0.868	0.3335	PFJA	0.143	0.5735	0.1665
TKA	0.312	0.77	0.332	TKA	0.3665	0.717	0.332

All tested models demonstrated F1 scores of greater than 0.7 in identifying radiograph series labelled by the EAP as optimal for a PKA; this indicates high capacity for recall and precision. The MobileNet v2, EfficientNet-B2, EfficientNet-ES, and DenseNet-121-xray models all achieved an F1 score of greater than 0.8 for the PKA classes. EfficientNet-ES was the best performing predictive model for a partial knee arthroplasty with AUC 95%, F1 score 83% and accuracy 79% (see Table 2).

## Discussion

This study confirms that a machine learning approach can closely replicate the ability of expert surgeons in identifying radiographically suitable patients for a medial or lateral partial knee arthroplasty. Indeed, the best performing algorithm (EfficientNet-ES) demonstrated an ‘excellent’ area under the curve (AUC) of 95%, F1 score of 83% and an accuracy of 79% in identifying partial knee replacement candidates [17]. Although not the primary aim of this study, the algorithm was similarly successful in identifying patients who would be unlikely to benefit from any arthroplasty surgery (AUC of 95%, F1 score of 70% and an accuracy of 88%), but less so in identifying patients for whom a total knee replacement (TKA) or patellofemoral joint replacement (PFJR) would be indicated.

A recent study by Houserman et al. [18], is the most relevant for comparison. They examined three X-ray views from 2767 patients, and trained an EffficientNetB4 algorithm with a 70:30 training: testing split. Interestingly, their algorithm outperformed ours, with an AUC of 96% and an accuracy of 89%. This was despite using only three radiographic views, compared to the four used in our study [19], and using treatment received as the ground truth rather than expert radiographic review, which would be expected to introduce more error. However, it should be noted that this algorithm only considered three treatment options (no arthroplasty, medial PKA, and TKA) which is a simpler task compared to the five outcomes predicted by our model. It also allowed for larger numbers in each group. Additionally, the case-mix was not comparable to our study, with 47% of their cohort not appropriate for arthroplasty, versus 16% in our group; this may reflect a stricter radiographic criterion for arthroplasty, or simply a different demographic of patients presenting to secondary care in the US.

With regards to radiographic atlas-based decision aids, The Knee Osteoarthritis Grading System (KOGS) [13], is most similar to the goals of our predictive algorithm given that it also includes five possible treatment outcomes (no arthroplasty, medial and lateral PKA, PFJA, and TKA). However, it utilises two extra radiographic views (six in total), and ground truth was based on treatment received. Its reliability in identifying PKAs, assessed by seven experienced surgeons reviewing 330 sets of X-rays, ranged from 92 to 98.8%, which is higher than the 80% accuracy achieved by our best performing algorithm. The main limitation being that the validation was performed by expert knee surgeons, and accuracy when used by low volume surgeons, or indeed by radiology or primary care doctors, is unknown.

Indeed, another atlas based radiological decision aid developed by Hamilton et al. [14], which only identifies patients suitable for a medial PKA, delivered an accuracy of 90% when 540 knees were assessed purely radiologically by one expert knee surgeon. However, a non-designer study of the same decision aid by 12 expert surgeons on 20 cases, reported significant variability, with the percentage of knees being identified as suitable for a PKA varying from 45 to 75%, translating to only a moderate statistical agreement (κ = 0.51).

It is reasonable to question whether 3D imaging, such as magnetic resonance imaging (MRI) might improve an algorithm’s ability to identify potential PKA candidates. However, not only does this have cost implications (the standard NHS MRI tariff is £110) [20], the evidence at present suggests that abnormal preoperative MRI findings do not affect the clinical outcomes of PKA selected using standard radiographic and clinical criteria [21].

There are of course limitations to this study. Numbers of cases can always be bigger, and it is reasonable to expect that this would improve algorithm performance. Opting for five possible treatment outcomes reduced the number of cases in each group for algorithm training, albeit this represents the main treatment decisions considered by compartmental knee surgeons. Testing was performed on 15% of the same data set used for training and validation, and so the applicability of the algorithm to external data, with different populations and X-ray protocols and machines is uncertain. Our algorithm was designed to be a radiographic screening tool of patients who might be suitable for a partial knee replacement, and it needs to be recognised that the final decision as to whether a patient is suitable for a PKA is based on a combination of history, clinical examination, imaging and patient values. Interestingly, other studies have used the final treatment as the ground truth for their radiographic analysis, whilst we believe such a tool would be best trained using multimodal data as above.

## Conclusions

In conclusion, we have developed a prototype algorithm capable of replicating expert surgeons in identifying patients suitable for either a medial or lateral partial knee arthroplasty (PKA). Arguably, it already has a clinically acceptable level of accuracy, but this could be expected to improve further with more data. There is an urgent need for this type of screening tool for patients with knee osteoarthritis, given the significant patient and socioeconomic benefits associated with PKA versus TKA. This was recognised in NICE’s 2020 guidelines which mandate that suitable patients should be offered a PKA, but joint registry data suggests that this is not happening in practice. Traditional radiographic atlas-based decision aids to help with this task have similarly failed to change practice, most likely because they are time consuming, open to subjective interpretation, and targeted at secondary care. The algorithm in this study has the potential to address this issue by automating the screening process in a rapid and objective manner, permitting results to be delivered at a primary care level, thereby empowering patients and primary care physicians to be involved in the treatment decision and referral to a surgical centre performing PKAs if needed.

Ultimately, this research finds clear evidence to support the application of artificial intelligence in providing diagnostic support for knee replacement. In particular, its ability to identify radiographically appropriate candidates for PKA is considered to have significant potential in addressing a major unmet need of current care pathways.
